# Locking and unlocking of quantum nonlocality without entanglement in local discrimination of quantum states

**DOI:** 10.1038/s41598-022-07930-w

**Published:** 2022-03-10

**Authors:** Donghoon Ha, Jeong San Kim

**Affiliations:** Department of Applied Mathematics and Institute of Natural Sciences, Kyung Hee University, Yongin, 17104 Republic of Korea

**Keywords:** Information theory and computation, Quantum physics

## Abstract

The phenomenon of nonlocality without entanglement (NLWE) arises in discriminating multi-party quantum separable states. Recently, it has been found that the post-measurement information about the prepared subensemble can lock or unlock NLWE in minimum-error discrimination of non-orthogonal separable states. Thus It is natural to ask whether the availability of the post-measurement information can influence on the occurrence of NLWE even in other state-discrimination strategies. Here, we show that the post-measurement information can be used to lock as well as unlock the occurrence of NLWE in terms of optimal unambiguous discrimination. Our results can provide a useful application for hiding or sharing information based on non-orthogonal separable states.

## Introduction

Quantum nonlocality is of central importance in multi-party quantum systems. A typical phenomenon of quantum nonlocality is quantum entanglement which is a useful resource for multi-party quantum communication^1^. Quantum entanglement is the correlation that cannot be shared among multiple parties using only *local operations and classical communication*(LOCC)^1–3^. However, it is also known that some nonlocal phenomena in multi-party quantum systems are still possible even in the absence of quantum entanglement.

*Nonlocality without entanglement*(NLWE) is another nonlocal phenomenon that arises in discriminating non-entangled states of multi-party quantum systems^4,5^. NLWE occurs when what can be achieved with global measurement in discriminating non-entangled states cannot be achieved only by LOCC. In the case of discriminating orthogonal non-entangled states, NLWE occurs when the perfect discrimination cannot be implemented by LOCC^5–9^. On the other hand, in the case of discriminating non-orthogonal non-entangled states, NLWE occurs when the globally optimal discriminations such as minimum-error discrimination^10–13^ or optimal unambiguous discrimination^14–17^ cannot be implemented by LOCC^18–21^. We also note that some non-local phenomena without entanglement can occur in the generalized probabilistic theories beyond quantum theory^22^.

In quantum state discrimination^23–26^, orthogonal states can be perfectly discriminated, whereas non-orthogonal states cannot. However, some non-orthogonal states can be perfectly discriminated when the post-measurement information about the prepared subensemble is available^27^. Nevertheless, some non-orthogonal states cannot be perfectly discriminated even the post-measurement information about the prepared subensemble is provided^28–30^. Therefore, in optimal discriminations with the post-measurement information about the prepared subensemble, the NLWE phenomenon arises when the globally optimal discrimination cannot be implemented by LOCC with the help of post-measurement information. Recently, it was shown that the availability of post-measurement information can lock or unlock NLWE in terms of minimum-error discrimination^31^, therefore it is natural to ask whether the post-measurement information affects the occurrence of NLWE in terms of state-discrimination strategies other than minimum-error discrimination.

Here, we show that even in optimal unambiguous discrimination, the availability of the post-measurement information about the prepared subensemble can affect the occurrence of NLWE. We first provide an ensemble of two-qubit product states having NLWE in terms of optimal unambiguous discrimination, and show that the availability of post-measurement information about the prepared subensemble vanishes the occurrence of NLWE, therefore *locking NLWE in terms of optimal unambiguous discrimination by post-measurement information*. We further provide another ensemble of two-qubit product state that does not have NLWE in terms of optimal unambiguous discrimination, and show that NLWE in the optimal unambiguous discrimination can be released when the post-measurement information about the prepared subensemble is provided. Thus *unlocking NLWE in terms of optimal unambiguous discrimination by post-measurement information*.

This paper is organized as follows. First, we present the form of two-qubit product state ensemble to be considered. In the “Methods” Section, we review the definitions and properties with respect to optimal unambiguous discrimination without and with post-measurement information and provide some useful lemmas in optimal local discrimination. As a main result of this paper, we provide a quantum state ensemble consisting of four two-qubit product states and show the occurrence of NLWE in terms of optimal unambiguous discrimination. With the same ensemble, we further show that NLWE does not occur in the optimal unambiguous discrimination with the post-measurement information about the prepared subensemble is available. As another main result of this paper, we provide another quantum state ensemble consisting of four two-qubit product states and show the non-occurrence of NLWE in terms of optimal unambiguous discrimination. With the same ensemble, we further show that NLWE occurs in the optimal unambiguous discrimination with the post-measurement information about the prepared subensemble.

## Results

Throughout this paper, we only consider the situation of unambiguously discriminating four states from the quantum state ensemble,

1 $$\begin{aligned} {\mathscr {E}}=\{\eta _{i},\rho _{i}\}_{i\in \Lambda },\ \Lambda =\{0,1,+,-\}, \end{aligned}$$

where $\rho _{i}$ is a $2\otimes 2$ non-entangled pure state,

2 $$\begin{aligned} \rho _{i}=|\varphi _{i}\rangle \!\langle \varphi _{i}| \ \ \text{ for } \text{ each } i\in \Lambda , \end{aligned}$$

and $\{|\varphi _{i}\rangle \}_{i\in \Lambda }$ is a product basis of ${\mathscr {H}}$. Each $\eta _{i}$ is the probability that the state $\rho _{i}$ is prepared.

The ensemble ${\mathscr {E}}$ can be seen as an ensemble consisting of two subensembles,

3 $$\begin{aligned} {\mathscr {E}}_{0}= & {} \{\eta _{i}/\sum _{j\in {\mathsf {A}}_{0}}\eta _{j},\rho _{i}\}_{i\in {\mathsf {A}}_{0}},\qquad {\mathsf {A}}_{0}=\{\,0\,,\,1\,\},\nonumber \\ {\mathscr {E}}_{1}= & {} \{\eta _{i}/\sum _{j\in {\mathsf {A}}_{1}}\eta _{j},\rho _{i}\}_{i\in {\mathsf {A}}_{1}},\qquad {\mathsf {A}}_{1}=\{+,-\}, \end{aligned}$$

where ${\mathscr {E}}_{0}$ and ${\mathscr {E}}_{1}$ are prepared with probabilities $\sum _{j\in {\mathsf {A}}_{0}}\eta _{j}$ and $\sum _{j\in {\mathsf {A}}_{1}}\eta _{j}$, respectively. The definitions and properties related to optimal unambiguous discrimination of ${\mathscr {E}}$ without and with post-measurement information are provided in the “Methods” Section.

Before we deliver our main results in the following subsections, we first provide the concepts of NLWE, NLWE with post-measurement information, and locking/unlocking NLWE by post-measurement information.

### **Definition 1**

For optimal unambiguous discrimination of a separable ensemble ${\mathscr {E}}$ in Eq. (1), NLWE occurs if and only if optimal unambiguous discrimination of ${\mathscr {E}}$ cannot be realized only by LOCC measurements, that is,

4 $$\begin{aligned} p_{\mathrm{L}}({\mathscr {E}})<p_{\mathrm{G}}({\mathscr {E}}). \end{aligned}$$

In discriminating orthogonal non-entangled states, NLWE occurs when the perfect discrimination cannot be realized by LOCC. Thus, the NLWE phenomenon of orthogonal non-entangled states is a special case of the NLWE phenomenon defined in Definition 1, that is, $p_{\mathrm{L}}({\mathscr {E}})<p_{\mathrm{G}}({\mathscr {E}})=1$. In the following definition, we provide the concept of NLWE in optimal unambiguous discrimination of ${\mathscr {E}}$ when the post-measurement information about the prepared subensemble is available.

### **Definition 2**

For optimal unambiguous discrimination of a separable ensemble ${\mathscr {E}}$ in Eq. (1) with post-measurement information $b\in \{0,1\}$ about the prepared subensemble ${\mathscr {E}}_{b}$ in Eq. (3), NLWE occurs if and only if optimal unambiguous discrimination of ${\mathscr {E}}$ with post-measurement information cannot be realized only by LOCC measurements, that is,

5 $$\begin{aligned} p_{\mathrm{L}}^{\mathrm{PI}}({\mathscr {E}})<p_{\mathrm{G}}^{\mathrm{PI}}({\mathscr {E}}). \end{aligned}$$

Now, we provide the concepts of *locking* and *unlocking* NLWE by post-measurement information in optimal unambiguous discrimination of ${\mathscr {E}}$.

### **Definition 3**

Let us consider the optimal unambiguous discrimination of a separable ensemble ${\mathscr {E}}$ in Eq. (1).

1. The post-measurement information $b\in \{0,1\}$ about the prepared subensemble ${\mathscr {E}}_{b}$ in Eq. (3) *locks* NLWE if NLWE occurs in discriminating the states of ${\mathscr {E}}$,
  6 $$\begin{aligned} p_{\mathrm{L}}({\mathscr {E}})<p_{\mathrm{G}}({\mathscr {E}}), \end{aligned}$$
  whereas NLWE does not occur when the post-measurement information *b* about the prepared subensemble is available,
  7 $$\begin{aligned} p_{\mathrm{L}}^{\mathrm{PI}}({\mathscr {E}})=p_{\mathrm{G}}^{\mathrm{PI}}({\mathscr {E}}). \end{aligned}$$
2. The post-measurement information *b* about the prepared subensemble ${\mathscr {E}}_{b}$ *unlocks* NLWE if NLWE does not occur in discriminating the states of ${\mathscr {E}}$,
  8 $$\begin{aligned} p_{\mathrm{L}}({\mathscr {E}})=p_{\mathrm{G}}({\mathscr {E}}), \end{aligned}$$
  whereas NLWE occurs when the post-measurement information *b* about the prepared subensemble is available,
  9 $$\begin{aligned} p_{\mathrm{L}}^{\mathrm{PI}}({\mathscr {E}})<p_{\mathrm{G}}^{\mathrm{PI}}({\mathscr {E}}). \end{aligned}$$

### Locking NLWE by post-measurement information in optimal unambiguous discrimination

In this section, we consider a situation where the post-measurement information about the prepared subensemble ${\mathscr {E}}_{b}$ *locks* NLWE in terms of optimal unambiguous discrimination. We first provide a specific example of a state ensemble ${\mathscr {E}}$ and show that NLWE in terms of optimal unambiguous discrimination occurs. With the same ensemble, we further show that the occurrence of NLWE in terms of optimal unambiguous discrimination can be vanished when post-measurement information is provided, thus locking NLWE by post-measurement information.

#### *Example 1*^31^

 Let us consider the ensemble ${\mathscr {E}}$ in Eq. (1) with

10 $$\begin{aligned} \begin{array}{lcllcllcl} \eta _{0}&{}=&{}\frac{\gamma }{2(1+\gamma )},&{} \rho _{0}&{}=&{}|\varphi _{0}\rangle \!\langle \varphi _{0}|,&{} |\varphi _{0}\rangle &{}=&{} |0\rangle \otimes |0\rangle ,\\ \eta _{1}&{}=&{}\frac{\gamma }{2(1+\gamma )},&{} \rho _{1}&{}=&{}|\varphi _{1}\rangle \!\langle \varphi _{1}|,&{} |\varphi _{1}\rangle &{}=&{} |0\rangle \otimes |1\rangle ,\\ \eta _{+}&{}=&{}\frac{1}{2(1+\gamma )},&{} \rho _{+}&{}=&{}|\varphi _{+}\rangle \!\langle \varphi _{+}|,&{} |\varphi _{+}\rangle &{}=&{} |+\rangle \otimes |+\rangle ,\\ \eta _{-}&{}=&{}\frac{1}{2(1+\gamma )},&{} \rho _{-}&{}=&{}|\varphi _{-}\rangle \!\langle \varphi _{-}|,&{} |\varphi _{-}\rangle &{}=&{} |-\rangle \otimes |-\rangle , \end{array} \end{aligned}$$

where $2\leqslant \gamma <\infty$, $\{|0\rangle ,|1\rangle \}$ is the standard basis in one-qubit system, and $|\pm \rangle =\frac{1}{\sqrt{2}}(|0\rangle \pm |1\rangle )$. In this case, the subensembles in Eq. (3) become

11 $$\begin{aligned} {\mathscr {E}}_{0}= & {} \{\frac{1}{2},|0\rangle \!\langle 0|\!\otimes \!|0\rangle \!\langle 0|,\ \frac{1}{2},|0\rangle \!\langle 0|\!\otimes \!|1\rangle \!\langle 1|\},\nonumber \\ {\mathscr {E}}_{1}= & {} \{\frac{1}{2},|+\rangle \!\langle +|\!\otimes \!|+\rangle \!\langle +|,\ \frac{1}{2},|-\rangle \!\langle -|\!\otimes \!|-\rangle \!\langle -|\}, \end{aligned}$$

with the probabilities of preparation $\frac{\gamma }{1+\gamma }$ and $\frac{1}{1+\gamma }$, respectively.

To show the occurrence of NLWE in terms of optimal unambiguous discrimination about the ensemble ${\mathscr {E}}$ in Example 1, we first evaluate the optimal success probability $p_{\mathrm{G}}({\mathscr {E}})$ defined in Eq. (48) of the “Methods” Section. The reciprocal vectors $\{|{\tilde{\varphi }}_{i}\rangle \}_{i\in \Lambda }$ corresponding to $\{|\varphi _{i}\rangle \}_{i\in \Lambda }$ defined in Eq. (10) are

12 $$\begin{aligned} |{\tilde{\varphi }}_{0}\rangle= & {} \sqrt{2}|\Phi _{-}\rangle ,\ |{\tilde{\varphi }}_{+}\rangle =\sqrt{2}|1+\rangle ,\nonumber \\ |{\tilde{\varphi }}_{1}\rangle= & {} \sqrt{2}|\Psi _{-}\rangle ,\ |{\tilde{\varphi }}_{-}\rangle =-\sqrt{2}|1-\rangle , \end{aligned}$$

where

13 $$\begin{aligned} |\Phi _{\pm }\rangle =\frac{1}{\sqrt{2}}|00\rangle \pm \frac{1}{\sqrt{2}}|11\rangle ,\ |\Psi _{\pm }\rangle =\frac{1}{\sqrt{2}}|01\rangle \pm \frac{1}{\sqrt{2}}|10\rangle . \end{aligned}$$

We can easily verify that the following $\{M_{i}\}_{i\in {\overline{\Lambda }}}$ is an unambiguous measurement satisfying the error-free condition in Eq. (47):

14 $$\begin{aligned} M_{0}= & {} |\Phi _{-}\rangle \!\langle \Phi _{-}|,\ M_{+}=0,\nonumber \\ M_{1}= & {} |\Psi _{-}\rangle \!\langle \Psi _{-}|,\ M_{-}=0,\nonumber \\ M_{?}= & {} \mathbbm {1}-|\Phi _{+}\rangle \!\langle \Phi _{+}| -|\Psi _{+}\rangle \!\langle \Psi _{+}|. \end{aligned}$$

Also, it is optimal because Condition (49) holds for this unambiguous measurement along with a positive-semidefinite operator

15 $$\begin{aligned} K=\frac{\gamma }{4(1+\gamma )}(|\Phi _{-}\rangle \!\langle \Phi _{-}| +|\Psi _{-}\rangle \!\langle \Psi _{-}|). \end{aligned}$$

Thus, the optimality of the measurement $\{M_{i}\}_{i\in {\overline{\Lambda }}}$ in Eq. (14) and the definition of $p_{\mathrm{G}}({\mathscr {E}})$ lead us to

16 $$\begin{aligned} p_{\mathrm{G}}({\mathscr {E}})=\mathrm {Tr}K=\frac{\gamma }{2(1+\gamma )}=\eta _{0}. \end{aligned}$$

In order to obtain the maximum success probability $p_{\mathrm{L}}({\mathscr {E}})$ defined in Eq. (51) of the “Methods” Section, we consider lower and upper bounds of $p_{\mathrm{L}}({\mathscr {E}})$. A lower bound of $p_{\mathrm{L}}({\mathscr {E}})$ can be obtained from the following unambiguous measurement $\{M_{i}\}_{i\in {\overline{\Lambda }}}$,

17 $$\begin{aligned} M_{0}= & {} 0,\ M_{+}=|1\rangle \!\langle 1|\otimes |+\rangle \!\langle +|,\nonumber \\ M_{1}= & {} 0,\ M_{-}=|1\rangle \!\langle 1|\otimes |-\rangle \!\langle -|,\nonumber \\ M_{?}= & {} |0\rangle \!\langle 0|\otimes (|+\rangle \!\langle +|+|-\rangle \!\langle -|), \end{aligned}$$

which can be implemented by finite-round LOCC because it can be realized by performing local measurements $\{|0\rangle \!\langle 0|,|1\rangle \!\langle 1|\}$ and $\{|+\rangle \!\langle +|,|-\rangle \!\langle -|\}$ on first and second subsystems, respectively. As we can easily verify that the success probability for the unambiguous LOCC measurement in Eq. (17) is $\frac{1}{2(1+\gamma )}$, the success probability is obviously a lower bound of $p_{\mathrm{L}}({\mathscr {E}})$,

18 $$\begin{aligned} p_{\mathrm{L}}({\mathscr {E}})\geqslant \frac{1}{2(1+\gamma )}=\eta _{+}. \end{aligned}$$

To obtain an upper bound of $p_{\mathrm{L}}({\mathscr {E}})$, let us consider a positive-semidefinite operator

19 $$\begin{aligned} H=\frac{1}{4(1+\gamma )}|1\rangle \!\langle 1|\otimes (|+\rangle \!\langle +|+|-\rangle \!\langle -|) \end{aligned}$$

with

20 $$\begin{aligned} \langle {\tilde{\varphi }}_{+}|H|{\tilde{\varphi }}_{+}\rangle =\eta _{+}=\eta _{-}=\langle {\tilde{\varphi }}_{-}|H|{\tilde{\varphi }}_{-}\rangle . \end{aligned}$$

Lemma 1 in the “Methods” Section leads us to

21 $$\begin{aligned} p_{\mathrm{L}}({\mathscr {E}})\leqslant \mathrm {Tr}H=\frac{1}{2(1+\gamma )}=\eta _{+}. \end{aligned}$$

Inequalities (18) and (21) imply

22 $$\begin{aligned} p_{\mathrm{L}}({\mathscr {E}})=\eta _{+}. \end{aligned}$$

From Eqs. (16) and (22), we note that there exists a nonzero gap between $p_{\mathrm{G}}({\mathscr {E}})$ and $p_{\mathrm{L}}({\mathscr {E}})$,

23 $$\begin{aligned} p_{\mathrm{L}}({\mathscr {E}})=\eta _{+}<\eta _{0}=p_{\mathrm{G}}({\mathscr {E}}), \end{aligned}$$

thus NLWE occurs in terms of optimal unambiguous discrimination in discriminating the states of the ensemble ${\mathscr {E}}$ in Example 1.

Now, we show that the availability of post-measurement information about the prepared subensemble vanishes the occurrence of NLWE in Inequality (23). To show it, we use the fact that the states of ${\mathscr {E}}$ in Example 1 can be unambiguously discriminated without inconclusive results using LOCC when the post-measurement information about the prepared subensemble is available^31^, or equivalently,

24 $$\begin{aligned} p_{\mathrm{L}}^{\mathrm{PI}}({\mathscr {E}})\geqslant 1. \end{aligned}$$

From the definitions of $p_{\mathrm{L}}^{\mathrm{PI}}({\mathscr {E}})$ and $p_{\mathrm{G}}^{\mathrm{PI}}({\mathscr {E}})$, we note that

25 $$\begin{aligned} p_{\mathrm{G}}^{\mathrm{PI}}({\mathscr {E}})\geqslant p_{\mathrm{L}}^{\mathrm{PI}}({\mathscr {E}}). \end{aligned}$$

As both $p_{\mathrm{G}}^{\mathrm{PI}}({\mathscr {E}})$ and $p_{\mathrm{L}}^{\mathrm{PI}}({\mathscr {E}})$ are bound above by 1, we have

26 $$\begin{aligned} p_{\mathrm{L}}^{\mathrm{PI}}({\mathscr {E}})=p_{\mathrm{G}}^{\mathrm{PI}}({\mathscr {E}})=1. \end{aligned}$$

Thus, NLWE does not occur in terms of optimal unambiguous discrimination in discriminating the states of the ensemble ${\mathscr {E}}$ in Example 1 when the post-measurement information about the prepared subensemble is available.

**Figure 1:**
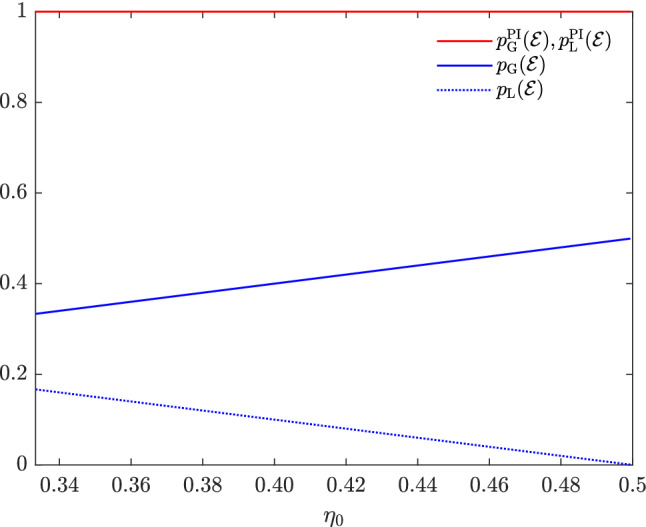
Locking NLWE by post-measurement information in terms of optimal unambiguous discrimination. For all $\eta _{0}\in [\frac{1}{3},\frac{1}{2})$, $p_{\mathrm{L}}({\mathscr {E}})$(dashed blue) is less than $p_{\mathrm{G}}({\mathscr {E}})$(solid blue), but $p_{\mathrm{L}}^{\mathrm{PI}}({\mathscr {E}})$(red) is equal to $p_{\mathrm{G}}^{\mathrm{PI}}({\mathscr {E}})$(red).

Inequality (23) shows that NLWE occurs in terms of optimal unambiguous discrimination about the ensemble ${\mathscr {E}}$ in Example 1, whereas Eq. (26) shows that NLWE does not occur when post-measurement information is available. Figure 1 illustrates the relative order of $p_{\mathrm{G}}({\mathscr {E}})$, $p_{\mathrm{L}}({\mathscr {E}})$, $p_{\mathrm{G}}^{\mathrm{PI}}({\mathscr {E}})$, and $p_{\mathrm{L}}^{\mathrm{PI}}({\mathscr {E}})$ for the range of $\frac{1}{3}\leqslant \eta _{0}<\frac{1}{2}$.

#### **Theorem 1**

*For optimal unambiguous discrimination of the ensemble* ${\mathscr {E}}$ *in Example* 1, *the post-measurement information about the prepared subensemble locks NLWE*.

### Unlocking NLWE by post-measurement information in optimal unambiguous discrimination

In this section, we consider the opposite situation to the previous section; the post-measurement information about the prepared subensemble ${\mathscr {E}}_{b}$ in Eq. (3) *unlocks* NLWE. After providing an example of a state ensemble ${\mathscr {E}}$, we first show that NLWE in terms of optimal unambiguous discrimination does not occur in discriminating the states of the ensemble. With the same ensemble, we further show the occurrence of NLWE in terms of optimal unambiguous discrimination in the state discrimination with the help of post-measurement information, thus unlocking NLWE by post-measurement information.

#### *Example 2*^31^

 Let us consider the ensemble ${\mathscr {E}}$ in Eq. (1) with

27 $$\begin{aligned} \begin{array}{lcllcllcl} \eta _{0}&{}=&{}\frac{\gamma }{2(1+\gamma )},&{} \rho _{0}&{}=&{}|\varphi _{0}\rangle \!\langle \varphi _{0}|,&{} |\varphi _{0}\rangle &{}=&{} |0\rangle \otimes |0\rangle ,\\ \eta _{1}&{}=&{}\frac{\gamma }{2(1+\gamma )},&{} \rho _{1}&{}=&{}|\varphi _{1}\rangle \!\langle \varphi _{1}|,&{} |\varphi _{1}\rangle &{}=&{} |0\rangle \otimes |1\rangle ,\\ \eta _{+}&{}=&{}\frac{1}{2(1+\gamma )},&{} \rho _{+}&{}=&{}|\varphi _{+}\rangle \!\langle \varphi _{+}|,&{} |\varphi _{+}\rangle &{}=&{} |+\rangle \otimes |+\rangle ,\\ \eta _{-}&{}=&{}\frac{1}{2(1+\gamma )},&{} \rho _{-}&{}=&{}|\varphi _{-}\rangle \!\langle \varphi _{-}|,&{} |\varphi _{-}\rangle &{}=&{} |+\rangle \otimes |-\rangle , \end{array} \end{aligned}$$

where $2\leqslant \gamma <\infty$. In this case, the subensembles in Eq. (3) become

28 $$\begin{aligned} {\mathscr {E}}_{0}= & {} \{\frac{1}{2},|0\rangle \!\langle 0|\!\otimes \!|0\rangle \!\langle 0|,\ \frac{1}{2},|0\rangle \!\langle 0|\!\otimes \!|1\rangle \!\langle 1|\}, \nonumber \\ {\mathscr {E}}_{1}= & {} \{\frac{1}{2},|+\rangle \!\langle +|\!\otimes \!|+\rangle \!\langle +|,\ \frac{1}{2},|+\rangle \!\langle +|\!\otimes \!|-\rangle \!\langle -|\}, \end{aligned}$$

with the probabilities of preparation $\frac{\gamma }{1+\gamma }$ and $\frac{1}{1+\gamma }$, respectively.

To show the non-occurrence of NLWE in terms of optimal unambiguous discrimination about the ensemble ${\mathscr {E}}$ in Example 2, we first evaluate the optimal success probability $p_{\mathrm{G}}({\mathscr {E}})$ defined in Eq. (48) of the “Methods” Section. Since the reciprocal vectors $\{|{\tilde{\varphi }}_{i}\rangle \}_{i\in \Lambda }$ corresponding to $\{|\varphi _{i}\rangle \}_{i\in \Lambda }$ defined in Eq. (27) are

29 $$\begin{aligned} |{\tilde{\varphi }}_{0}\rangle= & {} \sqrt{2}|-\rangle \otimes |0\rangle ,\ |{\tilde{\varphi }}_{+}\rangle =\sqrt{2}|1\rangle \otimes |+\rangle , \nonumber \\ |{\tilde{\varphi }}_{1}\rangle= & {} \sqrt{2}|-\rangle \otimes |1\rangle ,\ |{\tilde{\varphi }}_{-}\rangle =\sqrt{2}|1\rangle \otimes |-\rangle , \end{aligned}$$

the following measurement $\{M_{i}\}_{i\in {\overline{\Lambda }}}$ satisfies the error-free condition in Eq. (47),

30 $$\begin{aligned} M_{0}= & {} |-\rangle \!\langle -|\otimes |0\rangle \!\langle 0|,\ M_{+}=0,\nonumber \\ M_{1}= & {} |-\rangle \!\langle -|\otimes |1\rangle \!\langle 1|,\ M_{-}=0,\nonumber \\ M_{?}= & {} |+\rangle \!\langle +|\otimes (|0\rangle \!\langle 0|+|1\rangle \!\langle 1|). \end{aligned}$$

Moreover, the unambiguous measurement is optimal because Condition (49) holds for this unambiguous measurement along with the following positive-semidefinite operator

31 $$\begin{aligned} K=\frac{\gamma }{4(1+\gamma )}|-\rangle \!\langle -|\otimes (|0\rangle \!\langle 0|+|1\rangle \!\langle 1|). \end{aligned}$$

Thus, the optimality of the measurement $\{M_{i}\}_{i\in {\overline{\Lambda }}}$ in Eq. (30) and the definition of $p_{\mathrm{G}}({\mathscr {E}})$ lead us to

32 $$\begin{aligned} p_{\mathrm{G}}({\mathscr {E}})=\mathrm {Tr}K=\frac{\gamma }{2(1+\gamma )}=\eta _{0}. \end{aligned}$$

The measurement given in Eq. (30) can be performed using finite-round LOCC; two local measurements $\{|+\rangle \!\langle +|,|-\rangle \!\langle -|\}$ and $\{|0\rangle \!\langle 0|,|1\rangle \!\langle 1|\}$ are performed on first and second subsystems, respectively. Thus, the success probability for the unambiguous LOCC measurement in Eq. (30) is a lower bound of $p_{\mathrm{L}}({\mathscr {E}})$ defined in Eq. (51), therefore

33 $$\begin{aligned} p_{\mathrm{L}}({\mathscr {E}})\geqslant \eta _{0}, \end{aligned}$$

Moreover, from the definition of $p_{\mathrm{G}}({\mathscr {E}})$ and $p_{\mathrm{L}}({\mathscr {E}})$ in Eqs. (48) and (51), respectively, we have

34 $$\begin{aligned} p_{\mathrm{G}}({\mathscr {E}})\geqslant p_{\mathrm{L}}({\mathscr {E}}). \end{aligned}$$

Inequalities (33) and (34) lead us to

35 $$\begin{aligned} p_{\mathrm{L}}({\mathscr {E}})=p_{\mathrm{G}}({\mathscr {E}})=\eta _{0}. \end{aligned}$$

Thus, NLWE does not occur in terms of optimal unambiguous discrimination in discriminating the states of the ensemble ${\mathscr {E}}$ in Example 2.

Now, we show that NLWE in terms of optimal unambiguous discrimination occurs when the post-measurement information about the prepared subensemble is available. To show it, we use the fact that the states of ${\mathscr {E}}$ in Example 2 can be unambiguously discriminated without inconclusive results when the post-measurement information about the prepared subensemble is available^31^, or equivalently,

36 $$\begin{aligned} p_{\mathrm{G}}^{\mathrm{PI}}({\mathscr {E}})\geqslant 1. \end{aligned}$$

As $p_{\mathrm{G}}^{\mathrm{PI}}({\mathscr {E}})$ is bound above by 1, we have

37 $$\begin{aligned} p_{\mathrm{G}}^{\mathrm{PI}}({\mathscr {E}})=1. \end{aligned}$$

To obtain the maximum success probability $p_{\mathrm{L}}^{\mathrm{PI}}({\mathscr {E}})$ in Eq. (59) of the “Methods” Section, we consider lower and upper bounds of $p_{\mathrm{L}}^{\mathrm{PI}}({\mathscr {E}})$. For a lower bound of $p_{\mathrm{L}}^{\mathrm{PI}}({\mathscr {E}})$, let us first consider the following measurement $\{M_{\vec {\omega }}\}_{\vec {\omega }\in \Omega }$,

38 $$\begin{aligned} M_{(0,?)}= & {} |\nu _{-}\rangle \!\langle \nu _{-}|\otimes |0\rangle \!\langle 0|,\ M_{(?,+)}=|\nu _{+}\rangle \!\langle \nu _{+}|\otimes |+\rangle \!\langle +|, \nonumber \\ M_{(1,?)}= & {} |\nu _{-}\rangle \!\langle \nu _{-}|\otimes |1\rangle \!\langle 1|,\ M_{(?,-)}=|\nu _{+}\rangle \!\langle \nu _{+}|\otimes |-\rangle \!\langle -|, \nonumber \\ M_{\vec {\omega }}= & {} 0\ \forall \vec {\omega }\in \{(0,+),(0,-),(1,+),(1,-),(?,?)\}, \end{aligned}$$

where

39 $$\begin{aligned} |\nu _{\pm }\rangle =\sqrt{\frac{1}{2}\mp \frac{\gamma }{2\sqrt{1+\gamma ^{2}}}}|0\rangle \pm \sqrt{\frac{1}{2}\pm \frac{\gamma }{2\sqrt{1+\gamma ^{2}}}}|1\rangle . \end{aligned}$$

The measurement given in Eq. (38) is unambiguous because it satisfies the error-free condition in Eq. (55). Moreover, this measurement can be performed with finite-round LOCC; we first measure $\{|\nu _{+}\rangle \!\langle \nu _{+}|,|\nu _{-}\rangle \!\langle \nu _{-}|\}$ on first subsystem, and then measure $\{|+\rangle \!\langle +|,|-\rangle \!\langle -|\}$ or $\{|0\rangle \!\langle 0|,|1\rangle \!\langle 1|\}$ on second subsystem depending on the first measurement result $|\nu _{+}\rangle \!\langle \nu _{+}|$ or $|\nu _{-}\rangle \!\langle \nu _{-}|$. As we can verify from a straightforward calculation that the success probability for the unambiguous LOCC measurement in Eq. (38) is

40 $$\begin{aligned} \sum _{b\in \{0,1\}}\sum _{i\in {\mathsf {A}}_{b}} \eta _{i}\mathrm {Tr}\Big [\rho _{i} \sum _{\begin{array}{c} \vec {\omega }\in \Omega \\ \omega _{b}=i \end{array}} M_{\vec {\omega }}\Big ]= \sum _{i\in {\mathsf {A}}_{0}}\eta _{i}\mathrm {Tr}(\rho _{i}M_{(i,?)})+\sum _{j\in {\mathsf {A}}_{1}}\eta _{j}\mathrm {Tr}(\rho _{j}M_{(?,j)})=\frac{1}{2}\Big (1+\frac{\sqrt{1+\gamma ^{2}}}{1+\gamma }\Big ), \end{aligned}$$

thus the definition of $p_{\mathrm{L}}^{\mathrm{PI}}({\mathscr {E}})$ lead us to

41 $$\begin{aligned} p_{\mathrm{L}}^{\mathrm{PI}}({\mathscr {E}})\geqslant \frac{1}{2}\Big (1+\frac{\sqrt{1+\gamma ^{2}}}{1+\gamma }\Big ). \end{aligned}$$

We also note that the measurement in Eq. (38) yields $p_{\mathrm{guess}}({\mathscr {E}})$ defined in Eq. (62) of the “Methods” Section when considering $M_{0}=M_{(0,?)}$, $M_{1}=M_{(1,?)}$, $M_{+}=M_{(?,+)}$, and $M_{-}=M_{(?,-)}$^31^, that is,

42 $$\begin{aligned} p_{\mathrm{guess}}({\mathscr {E}})=\frac{1}{2}\Big (1+\frac{\sqrt{1+\gamma ^{2}}}{1+\gamma }\Big ). \end{aligned}$$

In order to obtain an upper bound of $p_{\mathrm{L}}^{\mathrm{PI}}({\mathscr {E}})$, let us consider the assumption of Lemma 2 in the “Methods” Section. For each $(\omega _{0},\omega _{1})\in {\mathsf {A}}_{0}\times {\mathsf {A}}_{1}$, there does not exist any nonzero product vector $|v\rangle =|a\rangle \otimes |b\rangle$ satisfying Condition (63); otherwise, $|a\rangle$ is not orthogonal to both $|0\rangle$ and $|+\rangle$. At the same time, $|b\rangle$ is orthogonal to the $|k\rangle$’s with $k\in \Lambda {\setminus }\{\omega _{0},\omega _{1}\}$, which leads us a contradiction. Thus, the guessing probability of ${\mathscr {E}}$ is also an upper bound of $p_{\mathrm{L}}^{\mathrm{PI}}({\mathscr {E}})$ due to Lemma 2 in the “Methods” Section, that is,

43 $$\begin{aligned} p_{\mathrm{L}}^{\mathrm{PI}}({\mathscr {E}})\leqslant p_{\mathrm{guess}}({\mathscr {E}})=\frac{1}{2}\Big (1+\frac{\sqrt{1+\gamma ^{2}}}{1+\gamma }\Big ). \end{aligned}$$

Inequalities (41) and (43) imply

44 $$\begin{aligned} p_{\mathrm{L}}^{\mathrm{PI}}({\mathscr {E}})=\frac{1}{2}\Big (1+\frac{\sqrt{1+\gamma ^{2}}}{1+\gamma }\Big ). \end{aligned}$$

From Eqs. (37) and (44), we note that there exists a nonzero gap between $p_{\mathrm{G}}^{\mathrm{PI}}({\mathscr {E}})$ and $p_{\mathrm{L}}^{\mathrm{PI}}({\mathscr {E}})$,

45 $$\begin{aligned} p_{\mathrm{L}}^{\mathrm{PI}}({\mathscr {E}}) =\frac{1}{2}\Big (1+\frac{\sqrt{1+\gamma ^{2}}}{1+\gamma }\Big ) <1=p_{\mathrm{G}}^{\mathrm{PI}}({\mathscr {E}}). \end{aligned}$$

Thus, NLWE occurs in terms of optimal unambiguous discrimination when the post-measurement information about the prepared subensemble is available.

**Figure 2:**
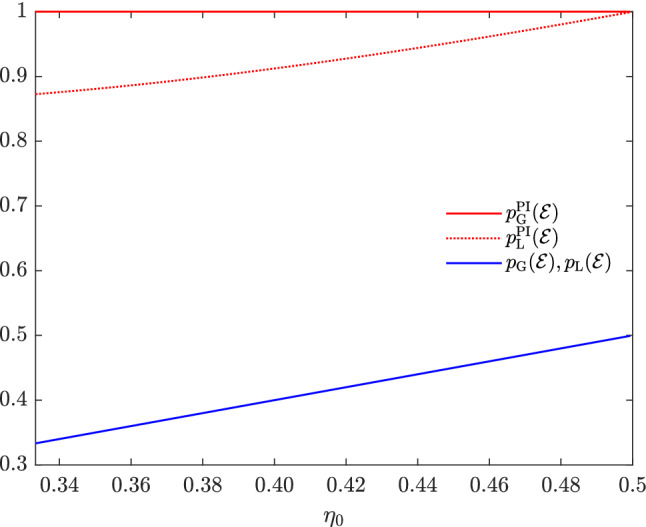
Unlocking NLWE by post-measurement information in terms of optimal unambiguous discrimination. For all $\eta _{0}\in [\frac{1}{3},\frac{1}{2})$, $p_{\mathrm{L}}({\mathscr {E}})$(blue) is equal to $p_{\mathrm{G}}({\mathscr {E}})$(blue), but $p_{\mathrm{L}}^{\mathrm{PI}}({\mathscr {E}})$(dashed red) is less than $p_{\mathrm{G}}^{\mathrm{PI}}({\mathscr {E}})$(solid red).

Equation (35) shows that NLWE in terms of optimal unambiguous discrimination does not occur in discriminating the states of the ensemble ${\mathscr {E}}$ in Example 2, whereas Inequality (45) shows that NLWE occurs when post-measurement information is available. Figure 2 illustrates the relative order of $p_{\mathrm{G}}({\mathscr {E}})$, $p_{\mathrm{L}}({\mathscr {E}})$, $p_{\mathrm{G}}^{\mathrm{PI}}({\mathscr {E}})$, and $p_{\mathrm{L}}^{\mathrm{PI}}({\mathscr {E}})$ for the range of $\frac{1}{3}\leqslant \eta _{0}<\frac{1}{2}$.

#### **Theorem 2**

*For optimal unambiguous discrimination of the ensemble* ${\mathscr {E}}$ *in Example* 2, *the post-measurement information about the prepared subensemble unlocks NLWE*.

## Discussion

We have shown that the post-measurement information about the prepared subensemble can lock or unlock NLWE in terms of optimal unambiguous discrimination. We have provided a quantum state ensemble consisting of four $2\otimes 2$ non-entangled pure states (Example 1) and shown the occurrence of NLWE in terms of optimal unambiguous discrimination with respect to the ensemble. With the same state ensemble, we have further shown that the availability of post-measurement information about the prepared subensemble vanishes the occurrence of NLWE, thus locking NLWE in terms of optimal unambiguous discrimination by post-measurement information (Theorem 1). Moreover, we have provided another quantum state ensemble consisting of four $2\otimes 2$ non-entangled pure states (Example 2) and shown the non-occurrence of NLWE in terms of optimal unambiguous discrimination with respect to the ensemble. With the same state ensemble, we have further shown the occurrence of NLWE in the optimal unambiguous discrimination with the post-measurement information about the prepared subensemble, thus unlocking NLWE in terms of optimal unambiguous discrimination by post-measurement information (Theorem 2).

We remark that the two state ensembles of this paper can also be used to demonstrate locking and unlocking NLWE in terms of minimum-error discrimination^31^. Thus, it is a natural future work to investigate locking and unlocking NLWE even in generalized state discrimination strategies such as an optimal discrimination with a fixed rate of inconclusive results^32–36^.

Our results can also provide us with a useful application in quantum cryptography. Whereas the existing quantum data hiding and secret sharing schemes are based on orthogonal states^37–41^, our results can extend those schemes to improved ones using non-orthogonal states. In Example 1, the availability of the post-measurement information about the prepared subensemble makes the globally hidden information accessible locally. On the other hand, in Example 2, the post-measurement information makes locally accessible information hidden locally but accessible globally. Finally, it is an interesting task to investigate if locking or unlocking NLWE by the post-measurement information about the prepared subensemble can depend on nonzero prior probabilities.

## Methods

In two-qubit (or $2\otimes 2$) systems, a state and a measurement are expressed by a density operator and a positive operator-valued measure(POVM), respectively, acting on a two-party complex Hilbert space ${\mathbb {C}}^{2}\otimes {\mathbb {C}}^{2}$. A density operator $\rho$ is a positive-semidefinite operator $\rho \succeq 0$ with unit trace $\mathrm {Tr}\rho =1$ and a POVM $\{M_{i}\}_{i}$ is a set of positive-semidefinite operators $M_{i}\succeq 0$ satisfying $\sum _{i}M_{i}=\mathbbm {1}$, where $\mathbbm {1}$ is the identity operator on ${\mathbb {C}}^{2}\otimes {\mathbb {C}}^{2}$. The probability of obtaining the measurement outcome corresponding to $M_{i}$ is $\mathrm {Tr}(\rho M_{i})$ when $\{M_{i}\}_{i}$ is performed on a quantum system prepared with $\rho$.

A positive-semidefinite operator is called *separable*(or *non-entangled*) if it is a sum of positive-semidefinite product operators; otherwise, it is said to be *entangled*. Also, a POVM is called *separable* if all elements are separable. In particular, a *LOCC measurement* that can be realized by LOCC is a separable measurement^2^.

### Optimal unambiguous discrimination

Let us consider the unambiguous discrimination of the states in ${\mathscr {E}}$ of Eq. (1) using a measurement $\{M_{i}\}_{i\in {\overline{\Lambda }}}$, where

46 $$\begin{aligned} {\overline{\Lambda }}=\Lambda \cup \{?\}=\{0,1,+,-,?\}. \end{aligned}$$

For each $i\in \Lambda$, $M_{i}$ is to detect $\rho _{i}$, and $M_{?}$ gives inconclusive results: “I don’t know what state is prepared.” The measurement $\{M_{i}\}_{i\in {\overline{\Lambda }}}$ can be expressed as

47 $$\begin{aligned} M_{i}=s_{i}|{\tilde{\varphi }}_{i}\rangle \!\langle {\tilde{\varphi }}_{i}|\ \ \forall i\in \Lambda ,\ M_{?}=\mathbbm {1}-\sum _{j\in \Lambda }s_{j}|{\tilde{\varphi }}_{j}\rangle \!\langle {\tilde{\varphi }}_{j}|, \end{aligned}$$

where $\{s_{i}\}_{i\in \Lambda }$ is a non-negative number set and $\{|{\tilde{\varphi }}_{i}\rangle \}_{i\in \Lambda }$ is the set of reciprocal vectors corresponding to $\{|\varphi _{i}\rangle \}_{i\in \Lambda }$ in Eq. (2) such that $\langle \varphi _{i}|{\tilde{\varphi }}_{j}\rangle =\delta _{ij}$^42^. We say a POVM $\{M_{i}\}_{i\in {\overline{\Lambda }}}$ is *unambiguous* if it satisfies the error-free condition in Eq. (47).

The *optimal unambiguous discrimination of* ${\mathscr {E}}$ is to minimize the probability of obtaining inconclusive results. Equivalently, the optimal unambiguous discrimination of ${\mathscr {E}}$ is to maximize the average probability of unambiguously discriminating states in ${\mathscr {E}}$;

48 $$\begin{aligned} p_{\mathrm{G}}({\mathscr {E}})=\max _{\mathrm{Eq.}(47)} \sum _{i\in \Lambda }\eta _{i}\mathrm {Tr}(\rho _{i}M_{i}) \end{aligned}$$

where the maximum is taken over all possible unambiguous measurements satisfying the error-free condition in Eq. (47). It is known that an unambiguous measurement $\{M_{i}\}_{i\in {\overline{\Lambda }}}$ is optimal if and only if there is a positive-semidefinite operator *K* satisfying the following condition^21,43–45^,

49 $$\begin{aligned} \begin{array}{c} \langle {\tilde{\varphi }}_{i}|K|{\tilde{\varphi }}_{i}\rangle \geqslant \eta _{i}\ \forall i\in \Lambda ,\ \mathrm {Tr}[M_{i}(K-\eta _{i}\rho _{i})]=0\ \forall i\in \Lambda ,\ \mathrm {Tr}(M_{?}K)=0. \end{array} \end{aligned}$$

In this case, we have

50 $$\begin{aligned} p_{\mathrm{G}}({\mathscr {E}})=\sum _{i\in \Lambda }\eta _{i}\mathrm {Tr}(\rho _{i}M_{i})=\mathrm{Tr}K \end{aligned}$$

if an unambiguous measurement $\{M_{i}\}_{i\in {\overline{\Lambda }}}$ and a positive-semidefinite operator *K* satisfy Condition (49)^21,43–45^.

When the available measurements are restricted to unambiguous LOCC measurements, we denote the maximum success probability by

51 $$\begin{aligned} p_{\mathrm{L}}({\mathscr {E}})=\max _{\begin{array}{c} \mathrm{Eq.}(47)\\ \mathrm{LOCC} \end{array}}\sum _{i\in \Lambda }\eta _{i}\mathrm {Tr}(\rho _{i}M_{i}). \end{aligned}$$

In the following lemma, we provide an upper bound of $p_{\mathrm{L}}({\mathscr {E}})$.

#### Lemma 1

*If H is a positive-semidefinite operator satisfying*

52 $$\begin{aligned} \langle {\tilde{\varphi }}_{i}|H|{\tilde{\varphi }}_{i}\rangle \geqslant \eta _{i} \end{aligned}$$

*for all reciprocal vectors* $|{\tilde{\varphi }}_{i}\rangle$ *that is a product vector, then* $\mathrm{Tr}H$ *is an upper bound of* $p_{\mathrm{L}}({\mathscr {E}})$.

#### Proof

Let us suppose that $\{M_{i}\}_{i\in {\overline{\Lambda }}}$ is an unambiguous LOCC measurement and $\chi$ is the set of all $i\in \Lambda$ such that $|{\tilde{\varphi }}_{i}\rangle$ is a product vector. Since every LOCC measurement is separable, $M_{i}$ is separable for all $i\in {\overline{\Lambda }}$. For all $i\in \Lambda$ with $i\notin \chi$, $M_{i}=0$ because $M_{i}$ is proportional to entangled $|{\tilde{\varphi }}_{i}\rangle \!\langle {\tilde{\varphi }}_{i}|$. Thus, the success probability is

53 $$\begin{aligned} \begin{array}{c} \sum _{i\in \chi }\eta _{i}\mathrm{Tr}(\rho _{i}M_{i}) \leqslant \sum _{i\in \chi }\eta _{i}\mathrm{Tr}(\rho _{i}M_{i}) +\sum _{i\in \chi }\mathrm{Tr}[(H-\eta _{i}\rho _{i})M_{i}]+\mathrm{Tr}(HM_{?}) =\mathrm{Tr}H, \end{array} \end{aligned}$$

where the inequality is due to the assumption of Inequality (52) and the positive-semidefiniteness of *H* and $M_{?}$, and the equality is from $M_{?}=\mathbbm {1}-\sum _{i\in \chi }M_{i}$. As Inequality (53) is true for any unambiguous LOCC measurement $\{M_{i}\}_{i\in {\overline{\Lambda }}}$, $\mathrm{Tr}H$ is an upper bound of $p_{\mathrm{L}}({\mathscr {E}})$. $\square$

### Optimal unambiguous discrimination with post-measurement information

Let us consider the situation of unambiguously discriminating the two-qubit states of ${\mathscr {E}}$ in Eq. (1) when the classical information $b\in \{0,1\}$ about the prepared subensemble ${\mathscr {E}}_{b}$ defined in Eq. (3) is given after performing a measurement. We use a POVM $\{M_{\vec {\omega }}\}_{\vec {\omega }\in \Omega }$ to unambiguously discriminate the states of ${\mathscr {E}}$ in Eq. (1), where $\Omega$ is the Cartesian product of two outcome sets ${\mathsf {A}}_{0}\cup \{?\}$ and ${\mathsf {A}}_{1}\cup \{?\}$ with inconclusive results,

54 $$\begin{aligned} \Omega= & {} \{(\omega _{0},\omega _{1})\,|\,\omega _{0}\in {\mathsf {A}}_{0}\cup \{?\},\, \omega _{1}\in {\mathsf {A}}_{1}\cup \{?\}\}\nonumber \\= & {} \{(0,+),(0,-),(1,+),(1,-),(0,?),(1,?),(?,+),(?,-),(?,?)\}. \end{aligned}$$

For each $(\omega _{0},\omega _{1})\in \Omega$, $M_{(\omega _{0},\omega _{1})}$ detects a state in ${\mathscr {E}}$ unambiguously or gives inconclusive results depending on post-measurement information $b\in \{0,1\}$. If $\omega _{b}\ne ?$, the state $\rho _{\omega _{b}}$ is detected unambiguously, that is, the POVM $\{M_{\vec {\omega }}\}_{\vec {\omega }\in \Omega }$ satisfies

55 $$\begin{aligned} \begin{array}{lll} \mathrm {Tr}[\rho _{-}M_{(i,+)}] =\mathrm {Tr}[\rho _{+}M_{(i,-)}] =0&{} \forall i\,\in {\mathsf {A}}_{0}\cup \{?\},\\ \mathrm {Tr}[\rho _{\,1}M_{(0,\,j)}] =\mathrm {Tr}[\rho _{\,0\,}M_{(1,\,j)}] =0&{} \forall j\in {\mathsf {A}}_{1}\cup \{?\}. \end{array} \end{aligned}$$

However, if $\omega _{b}=?$, inconclusive results are obtained. We say that a POVM $\{M_{\vec {\omega }}\}_{\vec {\omega }\in \Omega }$ is *unambiguous* if it satisfies the error-free condition in Eq. (55).

The *optimal unambiguous discrimination of* ${\mathscr {E}}$ *with post-measurement information* is to minimize the probability of obtaining inconclusive results. Equivalently, the optimal unambiguous discrimination of ${\mathscr {E}}$ with post-measurement information is to maximize the average probability of unambiguously discriminating states where the optimal success probability is defined as

56 $$\begin{aligned} p_{\mathrm{G}}^{\mathrm{PI}}({\mathscr {E}})= \max _{\mathrm{Eq.}(55)} \sum _{b\in \{0,1\}}\sum _{i\in {\mathsf {A}}_{b}} \eta _{i}\mathrm {Tr}\Big [\rho _{i} \sum _{\begin{array}{c} \vec {\omega }\in \Omega \\ \omega _{b}=i \end{array}} M_{\vec {\omega }}\Big ] \end{aligned}$$

over all possible unambiguous measurements in Eq. (55).

Rather surprisingly, some non-orthogonal states can be perfectly discriminated when the post-measurement information about the prepared subensemble is available^27^, that is, $p_{\mathrm{G}}^{\mathrm{PI}}({\mathscr {E}})=1$. More precisely, for a state ensemble ${\mathscr {E}}$ that consists of two subensembles ${\mathscr {E}}_{0}$ and ${\mathscr {E}}_{1}$ with two pure states, $p_{\mathrm{G}}^{\mathrm{PI}}({\mathscr {E}})=1$ if and only if

57 $$\begin{aligned} (1-G_{0+})(1-G_{1-})+(1-G_{0-})(1-G_{1+})-2\sqrt{G_{0+}G_{0-}G_{1+}G_{1-}}\geqslant 1, \end{aligned}$$

where

58 $$\begin{aligned} G_{ij}=\mathrm {Tr}(\rho _{i}\rho _{j}),\,i\in {\mathsf {A}}_{0},\,j\in {\mathsf {A}}_{1}. \end{aligned}$$

When the available measurements are limited to unambiguous LOCC measurements, we denote the maximum success probability by

59 $$\begin{aligned} p_{\mathrm{L}}^{\mathrm{PI}}({\mathscr {E}})= & {} \max _{\begin{array}{c} \mathrm{Eq.}(55)\\ \mathrm{LOCC} \end{array}} \sum _{b\in \{0,1\}}\sum _{i\in {\mathsf {A}}_{b}} \eta _{i}\mathrm {Tr}\Big [\rho _{i} \sum _{\begin{array}{c} \vec {\omega }\in \Omega \\ \omega _{b}=i \end{array}} M_{\vec {\omega }}\Big ]. \end{aligned}$$

We note that $p_{\mathrm{L}}^{\mathrm{PI}}({\mathscr {E}})$ in Eq. (59) can also be rewritten as

60 $$\begin{aligned} p_{\mathrm{L}}^{\mathrm{PI}}({\mathscr {E}}) =\max _{\begin{array}{c} \mathrm{Eq.}(55)\\ \mathrm{LOCC} \end{array}}\Bigg [\sum _{\vec {\omega }\in {\mathsf {A}}_{0}\times {\mathsf {A}}_{1}}{\tilde{\eta }}_{\vec {\omega }} \mathrm {Tr}({\tilde{\rho }}_{\vec {\omega }}M_{\vec {\omega }}) +\sum _{i\in {\mathsf {A}}_{0}}\eta _{i}\mathrm {Tr}(\rho _{i}M_{(i,?)})+\sum _{j\in {\mathsf {A}}_{1}}\eta _{j}\mathrm {Tr}(\rho _{j}M_{(?,j)}) \Bigg ], \end{aligned}$$

where

61 $$\begin{aligned} {\tilde{\eta }}_{\vec {\omega }} =\frac{1}{2}\sum _{b\in \{0,1\}}\eta _{w_{b}},\ {\tilde{\rho }}_{\vec {\omega }} =\frac{\sum _{b\in \{0,1\}}\eta _{w_{b}}\rho _{\omega _{b}}}{\sum _{b'\in \{0,1\}}\eta _{w_{b'}}}. \end{aligned}$$

We also note that Inequality (57) is a necessary but not sufficient condition for $p_{\mathrm{L}}^{\mathrm{PI}}({\mathscr {E}})=1$ because $p_{\mathrm{L}}^{\mathrm{PI}}({\mathscr {E}})=1$ implies $p_{\mathrm{G}}^{\mathrm{PI}}({\mathscr {E}})=1$ but not vice versa.

For an upper bound of $p_{\mathrm{L}}^{\mathrm{PI}}({\mathscr {E}})$, let us consider the following quantity,

62 $$\begin{aligned} p_{\mathrm{guess}}({\mathscr {E}})=\max _{\begin{array}{c} \{M_{i}\}_{i\in \Lambda }:\\ \mathrm{POVM} \end{array}}\sum _{i\in \Lambda }\eta _{i}\mathrm {Tr}(\rho _{i}M_{i}), \end{aligned}$$

which is the maximum average probability of correct guessing the prepared state when the available measurements are limited to LOCC measurements without inconclusive results^10–13^. The following lemma shows that $p_{\mathrm{guess}}({\mathscr {E}})$ can be used as an upper bound of $p_{\mathrm{L}}^{\mathrm{PI}}({\mathscr {E}})$.

#### Lemma 2

*For each* $(\omega _{0},\omega _{1})\in {\mathsf {A}}_{0}\times {\mathsf {A}}_{1}$, *if there is no nonzero product vector* $|v\rangle$ *satisfying*

63 $$\begin{aligned} \langle \varphi _{i}|v\rangle \ne 0\ \forall i\in \{\omega _{0},\omega _{1}\},\ \langle \varphi _{j}|v\rangle =0\ \forall j\in \Lambda {\setminus }\{\omega _{0},\omega _{1}\}, \end{aligned}$$

*then* $p_{\mathrm{guess}}({\mathscr {E}})$ *is an upper bound of* $p_{\mathrm{L}}^{\mathrm{PI}}({\mathscr {E}})$.

#### Proof

The assumption in (63) implies that for each $\vec {\omega }=(\omega _{0},\omega _{1})\!\in \!{\mathsf {A}}_{0}\!\times \!{\mathsf {A}}_{1}$, there does not exist any nonzero separable $M_{\vec {\omega }}\succeq 0$ that unambiguously detects the state $\rho _{\omega _{0}}$ or $\rho _{\omega _{1}}$ depending on post-measurement information $b=0$ or 1, respectively. Then, the term $\sum _{\vec {\omega }\in {\mathsf {A}}_{0}\times {\mathsf {A}}_{1}}{\tilde{\eta }}_{\vec {\omega }}\mathrm {Tr}({\tilde{\rho }}_{\vec {\omega }}M_{\vec {\omega }})$ in Eq. (59) disappears. Thus, we have

64 $$\begin{aligned} p_{\mathrm{L}}^{\mathrm{PI}}({\mathscr {E}})= & {} \max _{\begin{array}{c} \mathrm{Eq.}(55)\\ \mathrm{LOCC} \end{array}}\Bigg [\sum _{i\in {\mathsf {A}}_{0}}\eta _{i}\mathrm {Tr}(\rho _{i}M_{(i,?)}) +\sum _{j\in {\mathsf {A}}_{1}}\eta _{j}\mathrm {Tr}(\rho _{j}M_{(?,j)}) \Bigg ]\nonumber \\\le & {} \max _{\begin{array}{c} \{M_{i}\}_{i\in \Lambda }:\\ \mathrm{LOCC} \end{array}}\Bigg [\sum _{i\in {\mathsf {A}}_{0}}\eta _{i}\mathrm {Tr}(\rho _{i}M_{i}) +\sum _{j\in {\mathsf {A}}_{1}}\eta _{j}\mathrm {Tr}(\rho _{j}M_{j}) \Bigg ]=p_{\mathrm{guess}}({\mathscr {E}}), \end{aligned}$$

where the inequality is from the fact that $p_{\mathrm{guess}}({\mathscr {E}})$ is the maximum obtained from measurements without any constraint, whereas $p_{\mathrm{L}}^{\mathrm{PI}}({\mathscr {E}})$ is the maximum obtained from unambiguous LOCC measurements. $\square$
